# Mechanobiological dysregulation of the epidermis and dermis in skin disorders and in degeneration

**DOI:** 10.1111/jcmm.12060

**Published:** 2013-05-15

**Authors:** Rei Ogawa, Chao-Kai Hsu

**Affiliations:** a Department of Plastic, Reconstructive and Aesthetic Surgery, Nippon Medical SchoolTokyo, Japan; b Department of Dermatology, National Cheng-Kung University College of Medicine and HospitalTainan, Taiwan

**Keywords:** mechanobiology, mechanotransduction, skin, scar, keloid, dermis

## Abstract

During growth and development, the skin expands to cover the growing skeleton and soft tissues by constantly responding to the intrinsic forces of underlying skeletal growth as well as to the extrinsic mechanical forces from body movements and external supports. Mechanical forces can be perceived by two types of skin receptors: (1) cellular mechanoreceptors/mechanosensors, such as the cytoskeleton, cell adhesion molecules and mechanosensitive (MS) ion channels, and (2) sensory nerve fibres that produce the somatic sensation of mechanical force. Skin disorders in which there is an abnormality of collagen [*e.g*. Ehlers–Danlos syndrome (EDS)] or elastic (*e.g*. cutis laxa) fibres or a malfunction of cutaneous nerve fibres (*e.g*. neurofibroma, leprosy and diabetes mellitus) are also characterized to some extent by deficiencies in mechanobiological processes. Recent studies have shown that mechanotransduction is crucial for skin development, especially hemidesmosome maturation, which implies that the pathogenesis of skin disorders such as bullous pemphigoid is related to skin mechanobiology. Similarly, autoimmune diseases, including scleroderma and mixed connective tissue disease, and pathological scarring in the form of keloids and hypertrophic scars would seem to be clearly associated with the mechanobiological dysfunction of the skin. Finally, skin ageing can also be considered as a degenerative process associated with mechanobiological dysfunction. Clinically, a therapeutic strategy involving mechanoreceptors or MS nociceptor inhibition or acceleration together with a reduction or augmentation in the relevant mechanical forces is likely to be successful. The development of novel approaches such as these will allow the treatment of a broad range of cutaneous diseases.

- Introduction
- Skin disorders
  – Ehlers–Danlos syndrome (Cutis hyperelastica)
  – Cutis laxa
  – Scleroderma
  – Mixed connective tissue disease
  – Keloids and hypertrophic scars
  – Bullous pemphigoid
  – Neurofibromatosis
  – Leprosy
  – Diabetic skin ulcers
  – Lymphoedema
- Skin degeneration
  –Ageing
- Conclusion

## Introduction

Human skin is a complex organ that protects the body from the external environment, providing an effective barrier to external stimuli including water, gases and microbial invasion. During growth and development, the skin expands to cover the growing skeleton and soft tissues by constantly responding to the intrinsic forces of underlying skeletal growth as well as to the extrinsic forces from body movements and external mechanical stimuli. In a typical adult, the surface area of the skin is between 1.6 and 1.8 m^2^ 1.

Over the course of general cutaneous wound healing, granulation tissue, fibroblasts, myofibroblasts and endothelial as well as epithelial cells are subjected to intrinsic and extrinsic mechanical stimulation. The cutaneous wound itself contracts by the forces produced by myofibroblasts, in addition to being exposed to many extrinsic forces, including scratching, compression and the natural tension of the skin. Fibroblasts secrete collagen and fibronectin and regulate the volume of the extracellular matrix (ECM) by collagenase secretion. The sequential synthesis and enzymatic breakdown of proteins within the ECM result in the remodelling of three-dimensional ECM structures. Cell binding to matrix proteins exerts small forces that can cause cellular deformation. If the balance between ECM synthesis and degradation is not carefully maintained, scars, which are the final product of wound healing, may become hypertrophic or atrophic.

In addition to ECM alteration, the volume and flow of extracellular fluid (ECF) is increased in the wound according to the degree of blood vessel permeability. The ECF also exerts intrinsic mechanical forces, such as fluid shear force, hydrostatic pressure and osmotic pressure. These forces are perceived by two types of skin receptors 2: (1) cellular mechanoreceptors/mechanosensors, such as the cytoskeleton (*e.g*. actin filaments), cell adhesion molecules (*e.g*. integrin) and MS ion channels (*e.g*. Ca^2+^ channels) and (2) sensory nerve fibres (*e.g*. MS nociceptors) that produce the somatic sensation of mechanical force.

The mechanical forces generated by the ECM and ECF are important in maintaining the normal structure and function of the skin. Thus, mechanobiological dysregulation results in abnormal cutaneous wound healing, scarring and skin disorders (Table 1) in which there is an abnormality of collagen fibres [*e.g*. EDS] or elastic fibres (*e.g*. cutis laxa), or a malfunction of cutaneous nerve fibres (*e.g*. neurofibromatosis (NF) and diabetes mellitus). In addition, these disorders are characterized to some extent by deficiencies in mechanobiological functioning. Indeed, recent studies have shown that mechanotransduction is crucial for skin development 3, especially hemidesmosome maturation, which implies that the pathogenesis of skin disorders such as bullous pemphigoid is related to mechanobiology. Moreover, autoimmune diseases such as scleroderma, mixed connective tissue disease and pathological scarring, including keloid and hypertrophic scars, would seem to be clearly associated with the mechanobiological dysfunction of the skin. Skin ageing can also be considered as a degenerative process associated with mechanobiological dysfunction.

**Table 1:** Incidence rates of mechanobiology-related skin disorders

Disorder	Incidence
Ehlers–Danlos Syndrome (Cutis hyperelastica)	20/100,000 48
Cutis laxa	0.025/100,000 49
Scleroderma (systemic sclerosis)	100–200/100,000 50
Mixed connective tissue disease	0.1–0.3/100,000 51
Keloids	6–16% in African populations 52
Hypertrophic scars	40–70% following surgery 52
Bullous pemphigoid	7–14/100,000 53
Neurofibromatosis type I	33/100,000 54
Leprosy	250,000 cases detected globally every year 55
Diabetic skin ulcers	1–4.1% of 171 million diabetic patients globally 56
Lymphoedema	140–200 million cases globally 57

## Skin disorders

### Ehlers–Danlos syndrome (cutis hyperelastica)

The EDS is a heterogeneous group of inherited connective tissue disorders caused by genetic defects in the synthesis of type I, III, or V collagen 4. There are at least 10 recognized types of EDS, and the current classification (Villefranche, 1997) categorizes them into six subtypes (classic, hypermobility, vascular, kyphoscoliosis, arthrochalasis and dermatosparaxis) 5. The severity of the collagen gene mutations can vary from mild to life-threatening ones in their expression. Treatment of affected individuals is unsatisfactory and mainly supportive while requiring close monitoring of the digestive, the excretory, and particularly the cardiovascular systems. The therapeutic approaches, including physical therapy and corrective surgery, differ according to the particular disease manifestations.

Abnormal collagen leads to increased skin elasticity. Physical examination of the skin might reveal a velvety texture, fragility with easy tearing or bruising, redundant folds, molluscoid pseudotumours especially on pressure points, subcutaneous spheroids and fatty growth on the forearms or shins. Moreover, wound healing and scarring are abnormal, with widened (broad) and atrophic (thin) scars described as ‘cigarette paper’ scarring, easy bruising and a propensity for cutaneous bleeding 6, 7. Abnormal wound healing and delayed wound healing, both associated with EDS are because of deficiencies in dermal ECM-based mechanobiological processes that result in a decrease in collagen production. This sequence of events might contribute to the reduced amount of fibronectin production observed in the cultured fibroblasts of EDS patients compared to normal fibroblasts 8.

### Cutis laxa

While EDS is a deficiency of collagen fibres, cutis laxa is a deficiency of elastic fibres. The disease is inherited in most cases, but acquired forms are occasionally seen as well. Elastic fibres consist of elastin and play a fundamental role in skin structure and function 9. They are produced by fibroblasts and make up 2–5% of the dermis. As the dermal elastic network is a strong determinant of skin resilience, texture and quality 9, the state of the elastic fibres is closely related to the degree of cutaneous wrinkling and photoageing as well as to skin mechanobiology. Cutis laxa (also known as chalazoderma, dermatochalasia, dermatolysis, dermatomegaly, generalized elastolysis, generalized elastorrhexis and pachydermatocele) is a group of rare connective tissue disorders in which the skin becomes inelastic and hangs loosely in wrinkles and folds. The affected areas of skin may be thickened and dark. In addition, joints are loose (hypermobility) because of the lax ligaments and tendons 10. When cutis laxa is severe, it can also affect the internal organs, including a variety of severe impairments involving the lungs, heart, intestines and arteries 10. Patients also have cutaneous wound disruption and poor scarring, although cosmetic surgery procedures (face lifting) have been shown to be aesthetically and psychologically beneficial 11. The abnormal wound healing and scarring that occur in cutis laxa can also be considered dermal deficiencies in ECM-based mechanobiology.

### Scleroderma

Scleroderma (systemic sclerosis) is a complex systemic autoimmune disease (primarily of the skin) characterized by extensive fibrosis, vascular alterations and autoantibodies 12. There are two major subgroups: limited cutaneous scleroderma and diffuse cutaneous scleroderma. The cutaneous manifestations of the limited type mainly affect the hands, arms and face. It was previously called CREST syndrome in reference to the associated complications: calcinosis, Raynaud's phenomenon, oesophageal dysfunction, sclerodactyly and telangiectasias 13. In addition, pulmonary arterial hypertension may occur in up to one third of patients and is the most serious complication of this form of scleroderma. Diffuse scleroderma is rapidly progressive, involving a large area of the skin and one or more internal organs, frequently the kidneys, oesophagus, heart and lungs. This form of scleroderma can be quite disabling.

The overproduction of ECM components by fibroblasts, the principal effector cells, plays a key role in the pathogenesis of scleroderma 14. Reich *et al*. 14 investigated whether these functional alterations are accompanied by changes in the mechanical properties and morphology of fibroblasts. Atomic force microscopy was used to assess dermal fibroblasts derived either from scleroderma patients or from healthy donors. Significant differences in the cellular stiffness of dermal fibroblasts derived from sclerodermal lesions were detected. The authors thus concluded that the altered stiffness of sclerodermal fibroblasts may be important in disease pathogenesis as it could lead to an abnormal cellular response to mechanical stimuli. Another explanation for the overproduction of ECM is the activation of fibroblasts in the profibrotic cellular milieu by cytokines and growth factors, developmental pathways, endothelin 1 and thrombin 15. Factors including innate immune signalling *via* Toll-like receptors, matrix-generated biomechanical stress signalling *via* integrins, hypoxia and oxidative stress seem to be implicated in perpetuating the process 15. These data support a close association between scleroderma and mechanobiology.

### Mixed connective tissue disease

Mixed connective tissue disease, also known as Sharp's syndrome 16, combines the features of scleroderma, myositis, systemic lupus erythematosus and rheumatoid arthritis and is thus considered an overlap syndrome. It commonly causes joint pain/swelling, malaise, Raynaud phenomenon, Sjögren's syndrome, muscle inflammation and sclerodactyly (thickening of the skin of the finger pads) 16. As in scleroderma, matrix-generated biomechanical stress signalling *via* integrins is thought to be associated with the cutaneous chronic inflammation and delayed wound healing.

### Keloids and hypertrophic scars

Keloids and hypertrophic scars are fibroproliferative disorders of the skin, which in affected individuals appear red and show elevated scars with chronic inflammation at the affected sites. Several studies 17, 18 have determined a genetic predisposition, such as single nucleotide polymorphisms, to the development of keloids and hypertrophic scars 18. There is also increasing evidence that the local microenvironment plays a significant role 19, 20. Keloids show a marked preference for particular locations on the body and commonly adopt distinct site-specific shapes. These features have been shown to be highly dependent on local factors, especially mechanical force distribution 20. Thus, keloids usually occur at sites that are constantly or frequently subjected to mechanical forces (such as the anterior chest and scapular regions), but seldom occur in areas where stretching/contraction of the skin is rare (such as the parietal region or anterior lower leg), even in patients with multiple keloids. Moreover, the typical butterfly, crab's claw, or dumbbell shapes of keloids appear to be largely determined by the direction of the mechanical forces on and around the wound site 19. Thus, deficiencies in mechanosignalling pathways have been suggested to explain the formation of keloids and hypertrophic scars 21. As these lesions develop in the dermis, an understanding of dermal mechanobiology will be essential to unravelling their underlying mechanisms.

Moreover, hyperreactivity or derangement of the MS nociceptors of nerve fibres has been suggested to cause or contribute to the generation of keloid and hypertrophic scars 22. MS nociceptors on unmyelinated axons (C-fibres and Aδ-fibres) can be stimulated while the skin is stretched. In addition, axonal reflexes and the stimulation of antidromic sensory nerves result in the release of vasodilatory factors, including neuropeptides such as substance P and calcitonin gene-related peptide, which in turn induce local erythema. These neuropeptides may also up-regulate the expression of genes encoding growth factors, such as transforming growth factor β and nerve growth factor, in fibroblasts and other dermal cells. Therefore, neurogenic inflammation is a mediator that plausibly connects mechanical forces with the development of abnormal scar progression and/or generation.

### Bullous pemphigoid

This is an acute or chronic autoimmune skin disease involving a reaction against structural components of the hemidesmosome, resulting in subepidermal blistering, more appropriately known as bullae, within the space between the epidermis and dermis. Destruction of the hemidesmosomes is followed by the loss of epidermal adhesion and by the development of blisters between the keratinocyte layer and the dermis 23, 24.

Typically, there is a linear deposit of IgG at the basement membrane zone accompanied by the presence of circulating antibodies to BP230 and BP180 23. Iwata *et al*. 25 presented evidence that pathogenic autoantibodies against BP180 decrease the strength of keratinocyte adhesion to the cell matrix in the absence of complement and neutrophils 23.

Hemidesmosomes are very small stud- or rivet-like structures on the inner basal surface of epidermal keratinocytes. While desmosomes link two cells together, hemidesmosomes bind a cell to the ECM and thus play an important role in epidermal mechanobiology, specifically in cell-matrix adhesion 26. The breakdown of the epidermal mechanobiological environment is the main reason for the appearance of clinical symptoms.

### Neurofibromatosis

The NF is a genetically inherited disorder in which neurofibromas, which may be benign, may nonetheless cause serious damage by compressing nerves and other tissues. In NF type 1 (NF-1, also known as von Recklinghausen disease), unremarkable scars are produced after injury or surgery and progression to keloid or hypertrophic scar formation is rare 27. Miyawaki *et al*. 27 analysed 101 cases of NF or solitary neurofibroma recorded in centres from five different countries. The clinical diagnosis was NF-1 in 57 cases, solitary neurofibroma in 35, plexiform neurofibroma in four and no distinct clinical diagnosis in five. No patient in the NF group developed keloids or hypertrophic scars, whereas two patients in the solitary neurofibroma group had hypertrophic scarring 27. Given the obvious association between scar formation and mechanobiology 28, 29, the results of this study suggest that in NF the mechanobiological cutaneous environment is disrupted. Two possible pathways for this disruption have been suggested: (*i*) *via* neuropeptides or peripheral nerves and (*ii*) *via* the matrix.

An excess accumulation of neuropeptides is found in patients with pathological scarring 30. Hingtgen *et al*. 31 demonstrated that the capsaicin-stimulated release of neuropeptides is three- to fivefold higher in spinal cord slices from Nf1+/− mice than in wild-type mouse tissue. Extrapolated to NF-1 patients, while neuropeptides are produced in abundance, scarring is unremarkable. Nonetheless, controversy remains as to the underlying pathological mechanism. The absence of scarring in NF tissue can perhaps be explained by the fact that the disease instead develops in the deep dermis, 32 where the resulting structural changes may alter the cutaneous mechanobiology.

### Leprosy

Leprosy, also known as Hansen's disease, is a chronic condition caused by the bacteria *Mycobacterium leprae* and *Mycobacterium lepromatosis* 33. A loss of sensation and the destruction of the intra-epidermal innervation are the characteristic findings 34. An interesting feature of the disease is the absence of extensive scarring 34, which suggests that MS nociceptors involved in initiating the scarring process are denervated in these patients.

### Diabetic skin ulcers

Diabetes causes neuropathy, which inhibits nociception and the perception of pain. Consequently, diabetes patients often fail initially to notice small wounds involving the legs and feet and may therefore fail to prevent infection or repeated injury. Furthermore, diabetes causes immune compromise while the characteristic damage to small blood vessels prevents adequate tissue oxygenation, potentially resulting in chronic wounds. Pressure (compression) also plays a role in the formation of diabetic ulcers, as does infection. Regarding the mechanobiological aspects of the disease, fibronectin gene expression was shown to be enhanced in hypertrophic scars, but decreased in diabetic foot ulcers compared with normal skin 35. A deficiency of the MS nociceptors of nerve fibres has also been postulated as a cause or contributing factor in the generation of diabetic ulcers. Damage to sensory nerve fibres can lead to ‘neurogenic inflammation’, with the release of neuropeptides by sensory fibres and thus the accelerated production of cytokines by various activated cell types. Neurogenic inflammation is a form of mechanical stress-induced inflammation in which there is cutaneous antidromic vasodilatation and plasma extravasation. It is mediated by the release of neuropeptides from sensory nerve endings. Under normal conditions, the combined neurovascular control and neurogenic inflammation promote cutaneous wound healing, whereas this is not the case in diabetic patients 36.

### Lymphoedema

Lymphoedema, also known as lymphatic obstruction, is a condition of localized fluid retention and tissue swelling caused by a compromised lymphatic system. The lymphatic system returns the interstitial fluid to the thoracic duct and then to the bloodstream, where it is recirculated back to the tissues. In lymphoedema patients, the malfunction of this system results in an elevated risk of infection. Recently, an effective therapeutic approach consisting of lymphaticovenular anastomosis has been described 37.

Lymphatic fluid returns to the blood circulation through the force of osmosis acting on the venous capillaries. Prior to its return, the proteins, cellular debris and bacteria contained in the lymph are filtered through lymph collectors, which are blind-ended and epithelial-lined. Once the lymph enters the valved lymphatic vessels, the primary driving force for its movement is the rhythmic peristaltic-like pumping action of the smooth muscle cells lining the lymphatic vessel walls. A complex network of innervation regulates this system. Thus, lymphoedema can be considered a mechanobiological dysfunction involving osmosis and fluid pressure within the lymphatic system.

## Skin degeneration

### Ageing

Skin changes, including wrinkles, laxity and pigmentary irregularities, are among the most visible signs of ageing. The ageing process may be induced by environmental factors, such as sun exposure and smoking, or by chronological (intrinsic) factors 38, 39. The major feature of aged skin is the degeneration and fragmentation of the dermal collagen matrix, including collagen and elastic fibres (solar elastosis). Destruction of the ECM in aged skin further leads to the collapse of fibroblasts within the dermis, as these cells thereby no longer receive mechanical information. Consequently, collagen production is decreased while that of collagen-degrading enzymes (matrix metalloproteinases) is increased 40–42.

*In vivo* stimulation of *de novo* collagen production has been achieved by the injection of cross-linked hyaluronic acid dermal filler into photodamaged human skin 43, thereby restoring mechanical tension to the dermal matrix. At the surrounding sites of hyaluronic acid deposition, an increase in the number of fibroblasts has been demonstrated. Interestingly, these fibroblasts, which show a distinct, elongated stretched appearance, express high levels of type I procollagen together with an increased level of signalling molecules, such as transforming growth factor β and connective tissue growth factor 43, 44.

## Conclusion

Many of the mechanisms and clinical symptoms of skin disorders and skin degeneration are closely related to the mechanobiological environment *via* cellular components, ECM and the nervous system. A therapeutic strategy involving inhibition or activation of mechanoreceptors or MS nociceptors, together with reduction or augmentation in the relevant mechanical forces, is likely to be clinically successful. Mechanobiological intervention has been used to accelerate cutaneous wound healing 45, 46 and prevent pathological scarring 47. Novel agents or methods that reduce or augment mechanical force should facilitate the development of new therapeutic approaches for cutaneous diseases and skin ageing.

## References

[1] Bender AE, Bender DA (1995). Body surface area. A dictionary of food and nutrition.

[2] Ogawa R (2008). Keloid and hypertrophic scarring may result from a mechanoreceptor or mechanosensitive nociceptor disorder. Med Hypotheses.

[3] Zhang H, Landmann F, Zahreddine H (2011). A tension-induced mechanotransduction pathway promotes epithelial morphogenesis. Nature.

[4] De Paepe A, Malfait F (2012). The Ehlers-Danlos syndrome, a disorder with many faces. Clin Genet.

[5] Beighton P, De Paepe A (1988). Ehlers-Danlos syndromes: revised nosology, Villefranche, 1997. Ehlers-Danlos National Foundation (USA) and Ehlers-Danlos Support Group (UK). Am J Med Genet.

[6] Byers PH, Duvic M, Atkinson M (1997). Ehlers-Danlos syndrometype VIIA and VIIB result from splice-junction mutations or genomic deletions that involve exon 6 in the COL1A1 and COL1A2 genes of type I collagen. Am J Med Genet.

[7] Giunta C, Superti-Furga A, Spranger S (1999). Ehlers-Danlos syndrome type VII: clinical features and molecular defects. J Bone Joint Surg Am.

[8] Cutolo M, Castellani P, Borsi L (1986). Altered fibronectin distribution in cultured fibroblasts from patients with Ehlers-Danlos syndrome. Clin Exp Rheumatol.

[9] Rnjak J, Wise SG, Mithieux SM (2011). Severe burn injuries and the role of elastin in the design of dermal substitutes. Tissue Eng Part B.

[10] Berk DR, Bentley DD, Bayliss SJ (2012). Cutis laxa: a review. J Am Acad Dermatol.

[11] Thomas WO, Moses MH, Craver RD (1993). Congenital cutis laxa: a case report and review of loose skin syndromes. Ann Plast Surg.

[12] Gabrielli A, Avvedimento EV, Krieg T (2009). Scleroderma. N Engl J Med.

[13] Lauritano D, Bussolati A, Baldoni M (2011). Scleroderma and CREST syndrome: a case report in dentistry. Minerva Stomatol.

[14] Reich A, Meurer M, Eckes B (2009). Surface morphology and mechanical properties of fibroblasts from scleroderma patients. J Cell Mol Med.

[15] Bhattacharyya S, Wei J, Varga J (2011). Understanding fibrosis in systemic sclerosis: shifting paradigms, emerging opportunities. Nat Rev Rheumatol.

[16] Venables PJ (2006). Mixed connective tissue disease. Lupus.

[17] Marneros AG, Norris JE, Watanabe S (2004). Genome scans provide evidence for keloid susceptibility loci on chromosomes 2q23 and 7p11. J Invest Dermatol.

[18] Nakashima M, Chung S, Takahashi A (2010). A genome-wide association study identifies four susceptibility loci for keloid in the Japanese population. Nat Genet.

[19] Akaishi S, Akimoto M, Ogawa R (2008). The relationship between keloid growth pattern and stretching tension: visual analysis using the finite element method. Ann Plast Surg.

[20] Ogawa R, Okai K, Tokumura F (2012). The relationship between skin stretching/contraction and pathologic scarring: the important role of mechanical forces in keloid generation. Wound Repair Regen.

[21] Huang C, Akaishi S, Ogawa R (2012). Mechanosignaling pathways in cutaneous scarring. Arch Dermatol Res.

[22] Akaishi S, Ogawa R, Hyakusoku H (2008). Keloid and hypertrophic scar: neurogenic inflammation hypotheses. Med Hypotheses.

[23] Tsuruta D, Hashimoto T, Hamill KJ (2011). Hemidesmosomes and focal contact proteins: functions and cross-talk in keratinocytes, bullous diseases and wound healing. J Dermatol Sci.

[24] Sawamura D, Nakano H, Matsuzaki Y (2010). Overview of epidermolysis bullosa. J Dermatol.

[25] Iwata H, Kamio N, Aoyama Y (2009). IgG from patients with bullous pemphigoid depletes cultured keratinocytes of the 180-kDa bullous pemphigoid antigen (type XVII collagen) and weakens cell attachment. J Invest Dermatol.

[26] Selby JC (2011). Mechanobiology of epidermal keratinocytes: desmosomes, hemidesmosomes, keratin intermediate filaments, and blistering skin diseases. Mechanobiology of cell-cell and cell-matrix interactions.

[27] Miyawaki T, Billings B, Har-Shai Y (2007). Multicenter study of wound healing in neurofibromatosis and neurofibroma. J Craniofac Surg.

[28] Ogawa R (2011). Mechanobiology of scarring. Wound Repair Regen.

[29] Huang C, Ogawa R (2012). Fibroproliferative disorders and their mechanobiology. Connect Tissue Res.

[30] Scott JR, Muangman PR, Tamura RN (2005). Substance P levels and neutral endopeptidase activity in acute burn wounds and hypertrophic scar. Plast Reconstr Surg.

[31] Hingtgen CM, Roy SL, Clapp DW (2006). Stimulus-evoked release of neuropeptides is enhanced in sensory neurons from mice with a heterozygous mutation of the Nf1 gene. Neuroscience.

[32] Karvonen SL, Kallioinen M, Ylä-Outinen H (2000). Occult neurofibroma and increased S100 protein in the skin of patients with neurofibromatosis type 1: new insight to the etiopathomechanism of neurofibromas. Arch Dermatol.

[33] Sasaki S, Takeshita F, Okuda K (2001). Mycobacterium leprae and leprosy: a compendium. Microbiol Immunol.

[34] Facer P, Mann D, Mathur R (2000). Do nerve growth factor-related mechanisms contribute to loss of cutaneous nociception in leprosy?. Pain.

[35] Fu X, Yang Y, Sun T (2002). Comparative study of fibronectin gene expression in tissues from hypertrophic scars and diabetic foot ulcers. Chin Med Sci J.

[36] Schaper NC, Huijberts M, Pickwell K (2008). Neurovascular control and neurogenic inflammation in diabetes. Diabetes Metab Res Rev.

[37] Koshima I, Inagawa K, Urushibara K (2000). Supermicrosurgical lymphaticovenular anastomosis for the treatment of lymphedema in the upper extremities. J Reconstr Microsurg.

[38] Halder RM, Ara CJ (2003). Skin cancer and photoaging in ethnic skin. Dermatol Clin.

[39] Perner D, Vierkotter A, Sugiri D (2011). Association between sun-exposure, smoking behaviour and plasma antioxidant levels with the different manifestation of skin ageing signs between Japanese and German women – a pilot study. J Dermatol Sci.

[40] Fligiel SE, Varani J (2003). Collagen degradation in aged/photodamaged skin *in vivo* and after exposure to matrix metalloproteinase-1 *in vitro*. J Invest Dermatol.

[41] Varani J, Dame MK, Rittie L (2006). Decreased collagen production in chronologically aged skin: roles of age-dependent alteration in fibroblast function and defective mechanical stimulation. Am J Pathol.

[42] Varani J, Spearman D, Perone P (2001). Inhibition of type I procollagen synthesis by damaged collagen in photoaged skin and by collagenase-degraded collagen *in vitro*. Am J Pathol.

[43] Wang F, Garza LA, Kang S (2007). *In vivo* stimulation of de novo collagen production caused by cross-linked hyaluronic acid dermal filler injections in photodamaged human skin. Arch Dermatol.

[44] Fisher GJ, Varani J (2008). Looking older: fibroblast collapse and therapeutic implications. Arch Dermatol.

[45] Erba P, Ogawa R, Ackermann M (2011). Angiogenesis in wounds treated by microdeformational wound therapy. Ann Surg.

[46] Qureshi AA, Ross KM, Ogawa R (2011). Shock wave therapy in wound healing. Plast Reconstr Surg.

[47] Ogawa R, Akaishi S, Huang C (2011). Clinical applications of basic research that shows reducing skin tension could prevent and treat abnormal scarring: the importance of fascial/subcutaneous tensile reduction sutures and flap surgery for keloid and hypertrophic scar reconstruction. J Nippon Med Sch.

[48] Nienaber CA, Eagle KA (2003). Aortic dissection: new frontiers in diagnosis and management: Part I: from etiology to diagnostic strategies. Circulation.

[49] Maldergem VL, Dobyns W, Kornak U, Pagon RA, Bird TD, Dolan CR, Stephens K, Adam MP (1993). ATP6V0A2-related cutis laxa. GeneReviews™ [Internet].

[50] Barnes J, Mayes MD (2012). Epidemiology of systemic sclerosis: incidence, prevalence, survival, risk factors, malignancy, and environmental triggers. Curr Opin Rheumatol.

[51] Vinje O, Flatø B (2008). Epidemiologi ved blandet bindevevsykdom, mixed connective tissue disease (MCTD) hos barn. Norsk Epidemiologi.

[52] Gauglitz GG, Korting HC, Pavicic T (2011). Hypertrophic scarring and keloids: pathomechanisms and current and emerging treatment strategies. Mol Med.

[53] Stanley JR, Wolff K, Goldsmith LA, Katz SI, Gilchrest BA, Paller AS, Leffell DJ (2008). Bullous pemphigoid, chapter 54. Fitzpatrick's dermatology in general medicine.

[54] Friedman JM (1999). Epidemiology of neurofibromatosis type 1. Am J Med Genet.

[55] Rodrigues LC, Lockwood DNJ (2011). Leprosy now: epidemiology, progress, challenges, and research gaps. Lancet Infect Dis.

[56] Setacci C, de Donato G, Setacci F (2009). Diabetic patients: epidemiology and global impact. J Cardiovasc Surg.

[57] Cemal Y, Pusic A, Mehrara BJ (2011). Preventative measures for lymphedema: separating fact from fiction. J Am Coll Surg.

